# Can smartphones and tablets improve the management of childhood illness in Tanzania? A qualitative study from a primary health care worker’s perspective

**DOI:** 10.1186/s12913-015-0805-4

**Published:** 2015-04-02

**Authors:** Amani Flexson Shao, Clotilde Rambaud-Althaus, Ndeniria Swai, Judith Kahama-Maro, Blaise Genton, Valerie D’Acremont, Constanze Pfeiffer

**Affiliations:** Swiss Tropical and Public Health Institute, Basel, Switzerland; University of Basel, Basel, Switzerland; National Institute for Medical Research, Tukuyu Medical Research Center, Tukuyu, Tanzania; City Medical Office of Health, Dar es Salaam City Council, Tanzania; Infectious Diseases Service and Department of Ambulatory Care and Community Medicine, University Hospital, Lausanne, Switzerland

**Keywords:** Smartphones, Tablets, Electronic clinical decision support, IMCI, Primary care, Barriers, Electronic prescribing

## Abstract

**Background:**

The impact of the Integrated Management of Childhood Illness (IMCI) strategy has been less than anticipated because of poor uptake. Electronic algorithms have the potential to improve quality of health care in children. However, feasibility studies about the use of electronic protocols on mobile devices over time are limited. This study investigated constraining as well as facilitating factors that influence the uptake of a new electronic Algorithm for Management of Childhood Illness (ALMANACH) among primary health workers in Dar es Salaam, Tanzania.

**Methods:**

A qualitative approach was applied using in-depth interviews and focus group discussions with altogether 40 primary health care workers from 6 public primary health facilities in the three municipalities of Dar es Salaam, Tanzania. Health worker’s perceptions related to factors facilitating or constraining the uptake of the electronic ALMANACH were identified.

**Results:**

In general, the ALMANACH was assessed positively. The majority of the respondents felt comfortable to use the devices and stated that patient’s trust was not affected. Most health workers said that the ALMANACH simplified their work, reduced antibiotic prescription and gave correct classification and treatment for common causes of childhood illnesses.

Few HWs reported technical challenges using the devices and complained about having had difficulties in typing. Majority of the respondents stated that the devices increased the consultation duration compared to routine practice. In addition, health system barriers such as lack of staff, lack of medicine and lack of financial motivation were identified as key reasons for the low uptake of the devices.

**Conclusions:**

The ALMANACH built on electronic devices was perceived to be a powerful and useful tool. However, health system challenges influenced the uptake of the devices in the selected health facilities.

## Background

In the mid-1990s, the World Health Organization (WHO), the United Nations Children’s Fund (UNICEF) and other partners developed a strategy called ‘Integrated Management of Childhood Illnesses’ (IMCI) to improve the assessment, classification and treatment of the common causes of childhood mortality [1]. It was also aimed at improving health worker’s skills as well as family and community practices [2]. When used correctly, these guidelines were shown to improve quality of care and reduce the cost of treatment [3,4], and to reduce mortality in Tanzania [5].

However, the expected impact of IMCI worldwide has been less than anticipated due to limited uptake of the strategy [6]. The latter has been due to various challenges facing IMCI implementation at various levels as discussed in a study by Ahmed et al. [7]. For example, health workers (HWs) who are supposed to implement IMCI need about 11–16 days of training. The cost and time for this training has limited the uptake of IMCI worldwide [7]. In Tanzania, health workers’ compliance to IMCI algorithms is uneven and often quite low, both in rural and urban settings [4,8].

To address and overcome these challenges, new technologies are increasingly used, not only to facilitate training on IMCI [9] but also to improve compliance to evidence based guidelines [10-13]. In a pilot study, where HWs used personal digital assistance (PDAs) with an electronic version of IMCI (e-IMCI) in a rural dispensary in Mtwara, Southern Tanzania, DeRenzi et al. found that e-IMCI is as fast as routine practice (following IMCI from memory) and it increased compliance to the IMCI guidelines [10]. In another study by Mitchell et al. in Pwani region in Tanzania, HWs stated that it is much easier and faster to use electronic IMCI (e-IMCI) than paper based IMCI for consultations [13].

However, still little is known about facilitating factors and barriers and their influence on the long term uptake of electronic devices in the context of IMCI in developing countries. Using in-depth interviews and focus group discussions with primary health care workers, the present study aimed at providing insights into factors influencing the sustainable uptake of two different mobile technologies, tablets and smartphones, used as electronic decision support to implement a new algorithm aimed for rational use of antibiotics and antimalarials for the management of children in 6 health facilities (HF) in Dar es Salaam, Tanzania.

## Methods

### The PeDiAtrick project

The study was conducted within the PeDiAtrick project which aimed at improving the quality of healthcare for Tanzanian children by assessing the use of electronic decision support to promote evidence-based medicine and rational use of drugs. The project was implemented in Dar es Salaam, the largest city in Tanzania, and in Ifakara, a town in south-eastern Tanzania. In a first step, a paper as well as an electronic version of a new algorithm for management of childhood illness (ALMANACH) were developed (Rambaud-Althaus et al., submitted) and assessed in a safety study (Shao AF et al., submitted).

After the safety study had shown that the clinical outcome of patients was better using ALMANACH than with routine care, HWs from 3 selected HFs in Dar es Salaam received two days ALMANACH training by the field investigators (including first author) on the rational use of antibiotics and antimalarials using smartphones. Following the training each HW involved in taking care for children at the participating health facilities received face-to-face supervision by the field investigators (including first author) with several real patients onsite. In addition, user manuals on how to operate the smartphones were developed and given to each health facility for reference with training and face-to-face supervision. During the implementation of the smartphone study, the uptake and compliance to the algorithm by HWs was assessed (Rambaud-Althaus et al., submitted).

**Figure 1:**
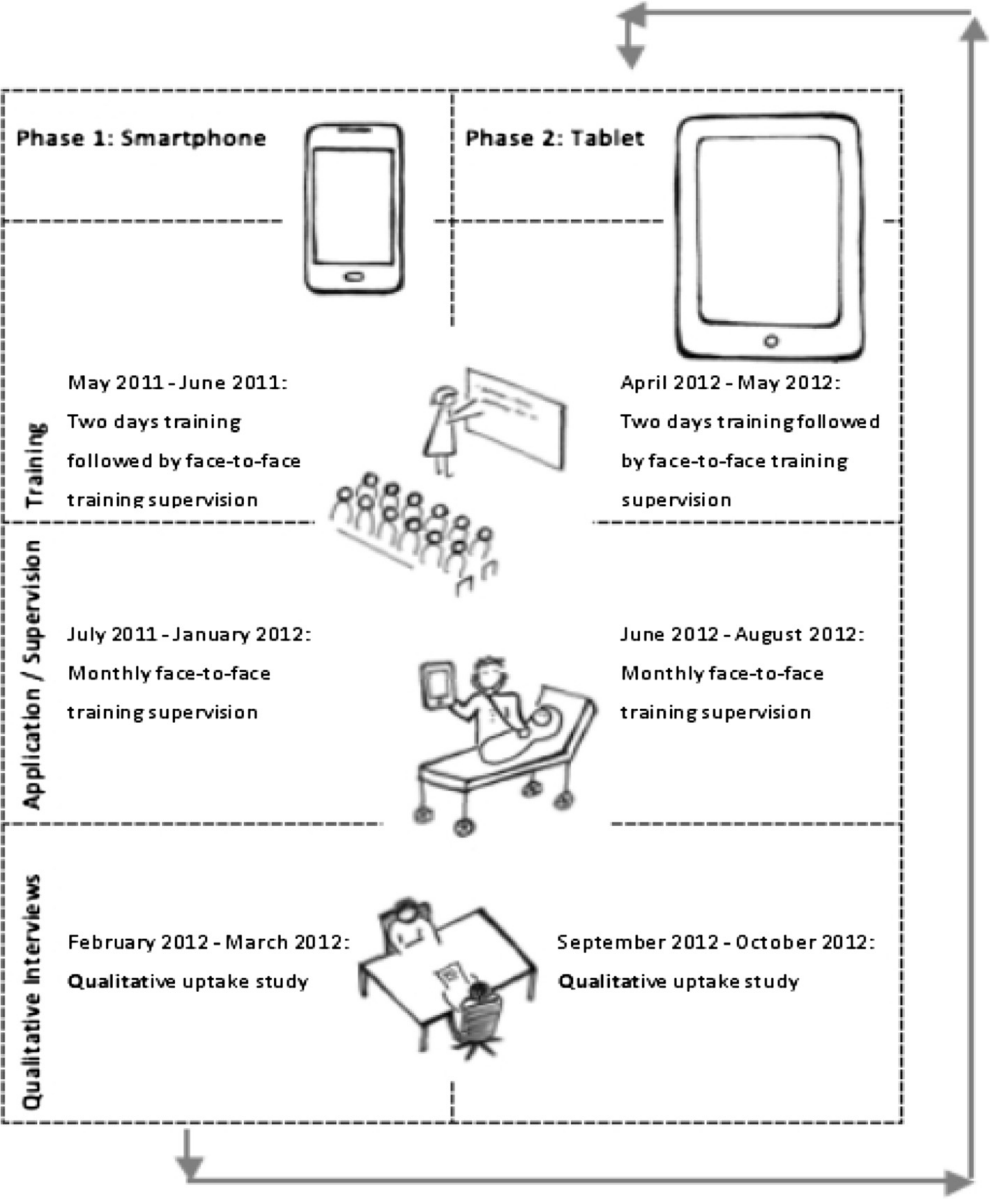
**Timeline of the smartphone/tablet introduction and qualitative study.**

**Figure 2:**
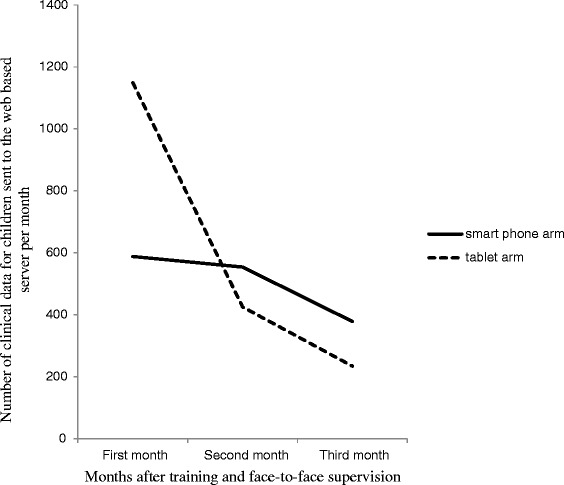
**Number of clinical data for children managed by HWs sent to the web based server in smartphone and tablet arms per month.**

The results from the smartphone arm were used to inform the design and introduction of tablets as electronic decision support for the management of similar group of sick children but in three other health facilities which were previously controls for the uptake and compliance study (Rambaud-Althaus et al., submitted). Figure 1 provides a summary of the intervention activities for the smartphones and tablets. Clinical data for each child managed by HWs using either smartphones or tables were sent to a web-based server. During the use of smartphones and tablets in the field over the course of three months, a decrease in the number of clinical data for children managed by HWs in both arms sent to the web based server was observed (Figure 2). The present study was thus designed to investigate the determinants of uptake and perception of health workers of these electronic devices to support clinical practice.

### Study design and setting

**Figure 3:**
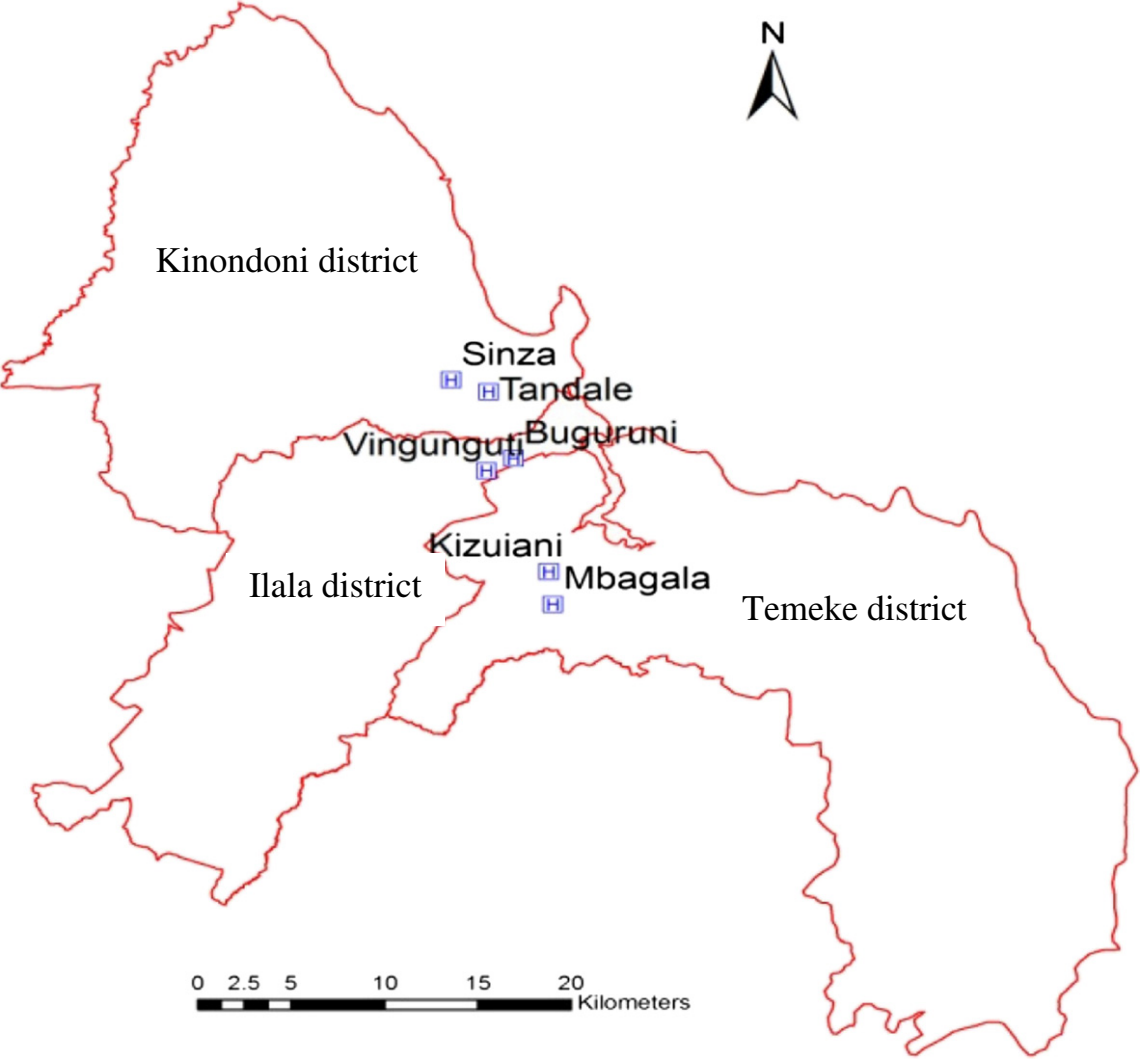
**Map of Dar es Salaam (including two selected health facilities in each district).**

The qualitative study presented here was carried out in the urban intervention sites in Dar es Salaam city in Tanzania between February and March 2012 (smartphones) and between September and October 2012 (tablets). Dar es Salaam was selected because it is a setting with moderate malaria endemicity [14] but high use of antibiotics [15]. The use of the new algorithm reduced unnecessary use of drugs both in low, moderate and high malaria endemic settings without exposing children to harm (Shao AF et al., submitted). Six health facilities (HF) were chosen from the three municipalities of Dar es Salaam, namely Ilala, Temeke and Kinondoni (Figure 3).

In each municipality, 2 public owned HF under the Ministry of Health and Social Welfare (MoHSW) which were involved in the uptake and compliance study (Rambaud-Althaus et al., submitted) were selected. Their characteristics are illustrated in Table 1.

**Table 1:** **Characteristics of the health facilities**

**Characteristic**	**Buguruni**	**Vingunguti**	**Mbagala**	**Kizuiani**	Sinza**	**Tandale**
**Maximun no of HWs**	**13**	**13**	**14**	**13**	**13**	**14**
Number of smartphones	4	N/A	5	N/A	N/A	3
Number of smartphones	4	N/A	5	N/A	N/A	3
Number of tablets	N/A	2	N/A	3	3	N/A
Maximum no of HWs in the morning shift at OPD	4	2-3	4-5	4	5	3
Daily attendance of patients*	200-250	120-150	400	150-200	500	300-500
Daily attendance of children <5 years*	50-60	40-50	70-100	60	160	100-150

Legend: OPD = Out Patient Department, N/A = Not Applicable.

*Numbers were provided by health facility in-charges in 2010.

**Was upgraded to a hospital in 2013.

HW = Health worker.

### Participant selection and data collection

Study participants were sampled among all clinicians who formed part of the project. In total 24 HWs (12 of the smartphone and 12 of the tablet arm) were purposely selected [16,17] using the following selection criteria: (1) 4 HWs per HF to represent each HF; (2) equal representation of 4 different uptake levels (for each of the four levels a median of the total number of patients recorded by the HWs for the first three months of implementation either by smartphones or by tablets was calculated): very low uptake (median number of cases: 2), low uptake (median 12), high uptake (median 35) and very high uptake (median 81).

The in-depth interviews were conducted by the first author, using a semi-structured and pilot tested interview guide in the local language, Kiswahili. The interviews were then transcribed in Kiswahili and translated into English by the first author. They took on average 44 minutes each (range 29–69 minutes). Only 1 HW from the smartphone arm refused to be tape-recorded but agreed to be interviewed. During this interview, detailed notes were taken.

In addition, at least 2 HWs from each of the 6 health facilities, who did not participate in the in-depth interviews, were invited to participate in focus group discussions. Three focus group discussions (FGD) were conducted, two from smartphone arm and one from tablet arm. Each FGD from the smartphone arm had 5 participants while the one from the tablet arm had 6 participants. Notes from the discussion were recorded by the first author and typed after completion of the discussion. All focus group discussions took about an hour.

### Data analysis

All in-depth interviews were recorded, transcribed, and translated from Swahili to English. Detailed notes taken during focus group discussions were translated into English. Data analysis was based on content analysis [18]. ATLAS.ti version 6.0 software was used to code interview transcripts for identification of common themes. Emerging themes were debated by a multidisciplinary team involved in the study. Data from both arms were compared to gain insights into similarities as well as differences of barriers and facilitating factors between smartphones and tablets related to the uptake of the new algorithm.

### Ethical consideration

The study was approved by the National Institute for Medical Research (NIMR) Review Board in Tanzania (NIMR/HQ/R.8a/Vol.IX/823). Approval was also provided by the institutional review boards of the University of Basel (EKBB) and the Ifakara Health Institute (IHI). All study participants provided written informed consent.

## Results

### Socio-demographic characteristics of the study participants

A total of 40 clinicians participated in the study, (24 in the in-depth interviews and 16 in the focus group discussions). For the in-depth interviews 24 clinicians were selected: 12 in the smartphone and 12 in the tablet arm. Of the 12 clinicians in the smartphone arm, 7 were females, 7 were aged below 40 years (range 24–55 years), 1 had smartphone experience and 1 had computer experience. Of the 12 clinicians in the tablet arm, 8 were females, 8 were aged below 40 years (range 27–58 years), none had smartphone experience while 4 had computer experience. For the focus group discussion, of 16 clinicians, 10 in the smartphone and 6 in the tablet arm. Of the 10 clinicians in the smartphones, 7 were females, 5 were aged below 40 years (range 27–53 years), none had smartphone experience while 2 had computer experience. Of the 6 clinicians in the tablet arm, 3 were females, 4 were aged below 40 years (range 30–48 years), none had smartphone experience while 1 had computer experience.

### Health care worker perceptions related to the application of smartphones and tablets

This section explores factors influencing the uptake of the two different devices, smartphone and tablet. Three themes emerged from in-depth interviews and focus group discussion data analysis. Because of similarities in themes between in-depth interviews and focus group discussions, results from both groups are presented together. The first theme describes HWs perceptions related to the application of the ALMANACH compared to routine practice. The second theme focuses around the technical usability of the device by HWs. The third theme looks at health system factors that were identified by HWs as key barriers to the long-term uptake of the ALMANACH.

**Figure 4:**
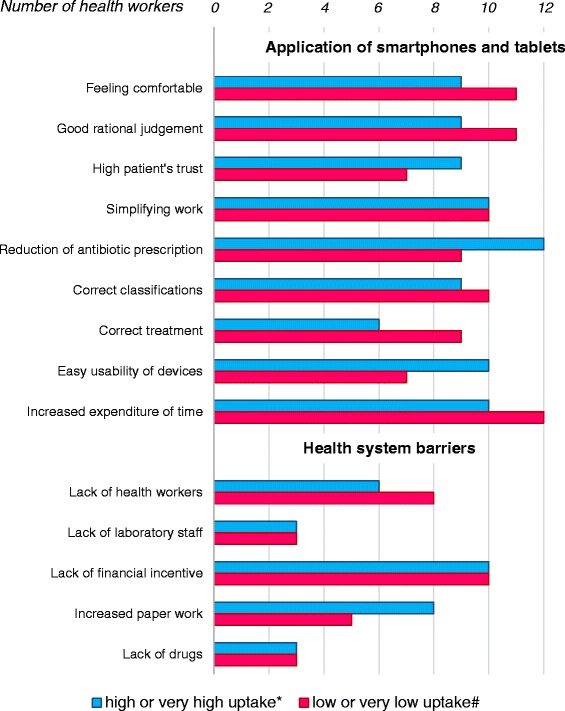
**Health workers’ perceptions stratified per ALMANACH uptake level.**

Figure 4 illustrates different presentations of these themes depending on the uptake level of respondents. Perceptions presented below were independent of the socio-demographic characteristics of the participants.

### Perceptions related to the application of smartphones and tablets

#### Feeling comfortable

Almost all HWs (12 smartphone/11 tablet) felt comfortable to use the electronic devices in front of the caretakers during consultation.

“*Of course I feel comfortable, not only because the tablet is a modern equipment, I know, I will not forget any symptoms related to disease. But sometimes […] you cannot remember everything, but by using ALMANACH you can tell this [child] has high respiration rate*”. (IDI, male, tablet, very high uptake)

#### Rational judgement

The majority of the study participants (9 smartphone/11 tablet) perceived that their rational judgment was not compromised by using the ALMANACH during consultations.

*“…the treatment provided by the tablet is short and clear, but there is also an opportunity to add other things […], it helps you to think more about the treatment*”. (IDI, female, tablet, low uptake)

#### Patient’s trust

Majority of the HWs, (8 smartphone/9 tablet) stated that patient’s trust was not affected by the use of electronic devices during consultations.

“*Because as I have told you that from my experience, most of them want to be treated by using the ALMANACH, they believe that something that is electronically is much better, I think that is the belief of our patients*”.

(IDI, male, tablet, very high uptake)

#### Simplifying work

The majority of the respondents (9 smartphone/11 tablet) said that smartphones and tablets simplified their work.

*“… for example […] if the patient is coughing, for each cough we gave antibiotics but through the phone you know this is pneumonia or this is normal chest cough so there is no need of using antibiotics. But another thing is that, it simplifies work because you are instructed to give medicine according to the weight of the child, so you don’t need to do the calculation of a dose*”. (IDI, male, smartphone, very low uptake)

#### Reduction of antibiotic prescription

Health workers pointed out that the ALMANACH assisted them to reduce antibiotic and antimalarial prescription as the device walked them step-by-step through the consultation starting from diagnosis to treatment including calculation of proper dosage of required drugs. Thus the majority of the study participants (10 smartphone/11 tablet) stated that both devices reduced antibiotic prescription compared to routine practice.

“*Yes, before I was prescribing antibiotics as antibiotics, I was just prescribing antibiotics, but truly now you don’t believe, now I know many diseases are febrile diseases, they don’t need antibiotics*”. (IDI, female, smartphone, very high uptake)

*“… if you are using ALMANACH the antibiotic consumption is reduced, if you don’t use ALMANACH the consumption of antibiotics is high*”. (IDI, female, tablet, very low uptake)

#### Correct classifications

The majority of study participants (9 smartphone/10 tablet) were quoted saying that both devices give correct classifications.

*“… so what was making me happy is that I used to get good diagnosis and good treatment”* (IDI, female, smartphone, high uptake)

*“Because if you are following the phone, it guides you directly, so if you make a good follow-up, you will get accurate diagnosis*”. (IDI, male, smartphone, low uptake)

#### Correct treatment

More than half of the respondents (8 smartphone/7 tablet) highlighted that the ALMANACH enabled correct treatment.

“*There are many advantages; first, the phone is a reference point in the sense that if you have forgotten what the patient is suffering from, or treatment or medication, by following the instructions in the phone you will know the diagnosis and medicine to that diagnosis. So the phone helps a lot*”. (IDI, male, smartphone, very low uptake)

### Usability of devices

#### Typing

In general, technical usability of the smartphone as well as tablet was considered to be easy. Few users (2 smartphone and 2 tablet) who all belong to low and very low uptake levels had difficulties typing, irrespective of their socio-demographic factors as well as computer/smartphone literacy.

“*This [typing] was difficult. This was a challenge for me. I was not familiar with the typing, it takes time to type.... I sometimes have to press and press, several times, it is difficult to use*.” (IDI, female, smartphone, very low uptake).

#### Length of ALMANACH

While the majority of health workers found both devices user-friendly, the vast majority (11 smartphones/10 tablet) were concerned about the length of the ALMANACH as it led to increased consultation duration. Few of the study participants (2 smartphones/4 tablet), apart from health system barriers, the high number of questions in the ALMANACH for the low uptake of the two devices. The average time for consultation duration using tablets was 8.7 minutes (range 2–38 minutes). The consultation duration for smartphones is not available because the smartphones arm involved a video component which was dropped in the subsequent evaluation of the tablets.

“*The biggest problem here is that we have few workers and a lot of patients, and if you see that you find it hard to ask a patient all those questions*”. (IDI, female, smartphone, high uptake)

### Health system barriers

#### Few health workers and many patients

Health system factors were identified as key barriers of the electronic ALMANACH uptake. More than half of the respondents (8 smartphone/6 tablet) expressed difficulties using the devices in an understaffed setting. Vice versa, the presence of many patients was regarded by the majority of HWs (8 smartphone/8 tablet) as a factor that prevented primary health care workers to use the two devices continuously. Thus the application of the devices was associated with over hours due to longer consultation periods. In addition, study participants were concerned about patients getting unhappy because of longer waiting times.

“*I feel free [to use the device] but the problem is the crowd of patients that we are supposed to attend, in our health facility we do not have enough staff, the use of phone is a barrier. The patients want to be treated and go back home early, so if they stay longer in the health facility, it becomes a problem*”. (IDI, Male, smartphone, very low uptake)

*“… Now it is a challenge to us as I am alone, so sometimes I cannot use phone*”. (IDI, Female, smartphone, very high uptake)

*“…if we had enough health workers, some for children and some for adults, then it could have helped a lot*”. (FGD, female, smartphone)

#### Few laboratory staff

Due to the use of the ALMANACH, more patients were sent to the laboratory compared to routine practice. Lack of laboratory personnel resulted in longer waiting hours for patients. In addition, clinicians had to wait for the patients whom they sent to the laboratory resulting in over hours. Few of the interviewed health workers (3 smartphone/3 tablet) stated that their concern about making patients wait negatively affected their uptake of the devices.

“*You find that our laboratory has many patients, so the patient stays in the queue for long time, waiting for the results*”. (IDI, female, tablet, very low uptake)

#### Lack of staff motivation

Lack of financial incentive was mentioned in almost all interviews. Majority of the participants (10 smartphone/10 tablet) expected financial compensation for using the devices at their respective health facilities. Participation in interventions or studies is often linked to financial benefits, and regarded as extra work that should be paid. They based their expectations on various reasons as narrated by a female health worker below:

“*They [the health workers] know there is no financial benefit, and they have conditioned themselves that phones waste time, knowing also that they do not get any income out of the phone use, that is the only reason when you come with this . . . not only the phone, when you come with books* [IMCI chart booklets]*, and one is working with children since morning, you find that they don’t even read them, they do not have any interest. They take the phones the same way they deal with books. The only difference is that this is a phone and that is a book, which you need to open*”. (IDI, female, smartphone, high uptake)

#### Increased paper work

Health workers in Tanzania are required to keep records about cases in the Tanzanian Health Management Information System (HMIS) also known in Kiswahili as Mfumo wa Taarifa za Huduma za Afya (MTUHA) [10]. During consultation, health personnel are supposed to keep assessment records in patients’ notebooks including date of consultation, diagnoses and treatment. However due to lack of training and time the uptake of these registers is poor [19]. More than half of the health workers (7 smartphones/7 tablets) attributed increased paper work as an extra work because besides having to use the electronic devices, the registers had to be filled too.

*“…yeah, it adds more work to us, because we have to enter the data in the phone, and then write the same data in the file, or patient’s card/notebook. In that way you do two things at the same time*”. (IDI, male, smartphone, very low uptake)

#### Lack of drugs

The application of the smartphone and tablet raised expectations among patients related to treatment that often could not be fulfilled. Few of the respondents (4 smartphone/2 tablet) expressed their frustration about lack of medicines in the health facility. After having walked their patients through the ALMANACH, treatment services were often regarded to be discouraging.

“*There is nothing which discourages you more as when you are using that tablet, and the caretaker has waited for such a long time, at the end you tell her, “go and buy the medicine outside or go to the medicine’s window” and there is no medicine, even the caretaker becomes discouraged, you have enrolled her in your tablet, and you have concentrated, at the end of the day, when she goes to the pharmacy she fails to get [the medicine]*”. (IDI, female, tablet, very low uptake)

“*When caretakers see the phones, they also expect that drugs are available*”. (FGD HW, male, smartphone)

## Discussion

To our best knowledge, this is the first qualitative study which investigated facilitating factors and barriers for routine use of electronic decision supports, namely smartphones and tablets, in developing countries.

Regardless of the type of device, results highlighted that the electronic ALMANACH was perceived to have various advantages over routine practice. Interestingly, the opinion on the two different tools, smartphone and tablet, was quite similar. Respondents talked mainly about the content of the ALMANACH itself, as well as context, and less on device related factors that influenced the uptake. Thus it is argued that the small technical differences between both tools did not impact on the uptake. Moxey et al. in a systematic review report that computerized clinical decision support systems with minimal threats to professional autonomy have a better chance of being accepted by users [20]. The ALMANACH offered health workers the freedom to select and type additional information [10].

The majority of the health workers both in high and low uptake levels felt comfortable to use smartphones and tablets in front of the caretakers for consultations. In addition, they stated that their judgement was not restricted and that caretakers’ trust was not affected. In a study using PDAs in the context of IMCI conducted in Tanzania, health workers were more skeptical about electronic decision support [13]. The different findings can be explained by the study design. The long-term follow up over several months including training and face-to-face supervision conducted in the present project contributed probably to the positive acceptance and evaluation of the devices. It is argued that long-term involvement is necessary to allow health workers to feel comfortable using mobile technology.

The majority of health workers concluded that the ALMANACH simplified their work, reduced the use of antibiotics, and assisted them to reach the correct diagnosis and treatment. Mitchell et al. also found that the use of PDAs lead to more accurate classifications compared to routine practice [21].

Considering this positive assessment, the rather low uptake of the ALMANACH over time is surprising and needs to be looked at more carefully. Hereby two reported interlinked barriers require further discussion: 1) the reported length of the ALMANACH as well as 2) health system barriers.

### Length of ALMANACH

To address the perceived excessive duration of consultation when using ALMANACH to manage patients, we are currently assessing the utility of each diagnostic pathway (branch) in the decision tree so that unnecessary questions can be discarded without jeopardizing safety and efficacy (Shao AF et al. 2014b in preparation). However, the length of the ALMANACH can only be shortened to a certain extent. In 2005, Tanzanian clinicians trained in paper IMCI took on average 8.2 minutes per child for a consultation [22]. In this study clinicians used on average 8.7 minutes (2–38 minutes) per child for consultation with tablets. Findings from another study [13] conducted in a rural setting in Pwani Region in Tanzania, which highlighted that it is faster to use electronic protocols than IMCI chart booklet, could not be confirmed. Instead results from urban Dar es Salaam showed that electronic clinical decision support systems lead to more laboratory tests done compared to routine practice [20] resulting in longer consultation duration and more laboratory work. However, it must be considered that consultation time varies depending on the setting. In rural areas it is often longer than in urban ones. Earlier studies showed that the shorter the consultation duration, the higher the risk that children are not properly assessed [23,24]. This can lead to poor clinical outcomes especially when danger signs are not properly identified. Case management and health outcomes cannot be improved if clinicians are not prepared to allocate sufficient time for consultations following IMCI guidelines [24]. The willingness to follow medical standards is a pre-requisite for the sustainable application not only of electronic tools such as ALMANACH but also decision charts in general, whatever the support.

### Health system barriers

Although the majority of the respondents assessed the devices very positively and highlighted their strengths, they also reported health system related barriers in almost all interviews. The Tanzanian health system is characterized by shortage of health care providers, which affects also intervention aimed at improving health care delivery [25-27]. Understaffing and high number of patients prevented many clinicians from making long-term use of the electronic devices as they were concerned that these new tools lead to excessive delays for patients and over hours of health workers. This may be the reality in some places, but it may also be due to health workers’ perception and beliefs. An assessment of health workers performance during outpatient consultations in Dodoma (urban) and Morogoro region (rural) reported that clinicians have ample time and are not overworked [28]. On the other hand, the same researchers mention that there could be a relationship between low performance of clinicians and high workload [28].

Health care workers complained about recording clinical records for patients in both the mobile tools and the clinical registry books (MTUHA) required by the Tanzanian HMIS. This was perceived as double work. However, this duplication was related to the nature of this pilot implementation where routine data collected through the electronic devices directly to the server were not considered suitable for the continuous monitoring of diagnoses and treatment, because they could not be linked to the usual HMIS. This concern would ultimately no more be relevant, once all clinical data can be collected directly in an integrated electronic monitoring system.

Lack of financial incentive was an additional barrier mentioned by the interviewees. Health workers perceived the use of the electronic tools as extra work, and expected therefore extra payment. No incentives were given during field implementation because the smartphone or tablet were designed and used as an aid to appropriately assess a sick child, and not as a research tool to satisfy scientific curiosity. The same procedure was applied when Rapid Diagnostic Tests for malaria were pilot-implemented in Dar es Salaam, and the intervention went smoothly and was then scaled up in a national strategy [15].This is of course a risk since previous studies reported poor performance of health personnel when there was no monetary or non-monetary incentives [28-31]. It is common practice to pay incentives when extra work is asked to the clinician and no benefit to either staff or patients is expected. In the present study, the use of smartphones and tablets was aimed at improving clinical outcome and reducing unnecessary antibiotic prescriptions. As a result, mechanisms for clinicians to get informal payments such as by running “private” drug shops (selling medicines within the consultation room) [32] were not possible. Therefore it is possible that the absence of incentives for what could be considered as extra work because of the research environment, and the potential reduction of informal payments because of smartphone or tablet use, could have contributed to their poor uptake.

Previous studies reported that interventions which interrupt or slowdown patients’ flow can lead to uptake challenges [33,34]. This was confirmed by the participants who complained about a slowdown caused by the use of the ALMANACH. Patients assessed using the electronic devices were more likely to be sent to the laboratory for testing.

The way respondents presented health system barriers was in most of the cases the same regardless of the uptake levels. Belonging to a low uptake level was therefore not linked to a negative assessment of the device. The very same health personnel with low uptake level that praised the ALMANACH, highlighted the impact of health system barriers and held them responsible for the low uptake. In a nutshell, health system barriers countervailed the positive evaluation and continued use of the ALMANACH.

Sufficient health workforce, good access to medical products and technologies, effective service delivery systems are crucial for achieving good quality of care [35]. Results of this study highlight that sufficient staff, adequate payment system, enabling working environments including supportive supervision, and access to affordable medicine are important factors that influenced the uptake of the ALMANACH. However, increasing, staffing, training and supervision might not be sufficient [28,36]. Additional factors such as the individuals’ motivations or how much energy the individual staffs decide to invest in improving the quality of health care, are necessary for enhancing the quality of health care. These factors are influenced by other determinants such as individual’s ethical and medical standards [28].

### Study limitations

The study focused on primary health care workers in the largest city of Tanzania, Dar es Salaam. While the results might be applicable to other urban settings, they cannot be generalized to rural settings that are often characterized by less patients and different health care infrastructure.

The role of the first author which included project implementation as well as data collection might have influenced the responses of the study participants. In order to deal with that, all study participants were encouraged to be as open as possible prior to the interview. In addition, participants were assured that the interviews aimed at assessing the ALMANACH and factors affecting its implementation following the low uptake observed. The interviews showed that respondents felt at ease sharing their experiences with the first author as they had over the years developed trust and mutual respect.

Although the caretaker’s perspectives were not considered in the study, high proportion of health personnel spoke about the positive reactions of caretakers and concluded that their trust was not affected.

It is argued that future studies should not only focus on technical aspects of mobile technology. Context related aspects needs to be addressed as well in order to foster a setting that allows for sustainable uptake and use of mobile devices.

## Conclusions

The present study contributed to the increasing body of mobile health literature from the perspective of primary care health workers. Findings show that smartphones as well as tablets using ALMANACH have the potential to improve the management of childhood illness. The majority of the health workers had no technical problems using the two electronic devices (smartphones and tablets) with the ALMANACH. Both were perceived as powerful tools which simplify work, give correct classification and reduce unnecessary use of antibiotics. However, health system barriers influence the long-term uptake. To ensure sustainable uptake of mHealth interventions such as ALMANACH, contextual barriers identified in this study should be addressed. Further studies are needed in order to better understand whether uptake is feasible for health workers given health system barriers and additional contextual factors. The ALMANACH presented in this paper will be improved and further tested in similar patients population in resource poor settings. Hereby barriers highlighted by health workers s will be addressed.

## References

[1] Gove S (1997). Integrated management of childhood illness by outpatient health workers: technical basis and overview. The WHO Working Group on Guidelines for Integrated Management of the Sick Child. Bull World Health Organ.

[2] Lambrechts T, Bryce J, Orinda V (1999). Integrated management of childhood illness: a summary of first experiences. Bull World Health Organ.

[3] Bryce J, Gouws E, Adam T, Black RE, Schellenberg JA, Manzi F, Victora CG, Habicht JP (2005). Improving quality and efficiency of facility-based child health care through Integrated Management of Childhood Illness in Tanzania. Health Policy Plan.

[4] Armstrong Schellenberg J, Bryce J, de Savigny D, Lambrechts T, Mbuya C, Mgalula L, Wilczynska K (2004). The effect of Integrated Management of Childhood Illness on observed quality of care of under-fives in rural Tanzania. Health Policy Plan.

[5] Armstrong Schellenberg JR, Adam T, Mshinda H, Masanja H, Kabadi G, Mukasa O, John T, Charles S, Nathan R, Wilczynska K (2004). Effectiveness and cost of facility-based Integrated Management of Childhood Illness (IMCI) in Tanzania. Lancet.

[6] Victora CG, Huicho L, Amaral JJ, Armstrong-Schellenberg J, Manzi F, Mason E, Scherpbier R (2006). Are health interventions implemented where they are most needed? District uptake of the integrated management of childhood illness strategy in Brazil, Peru and the United Republic of Tanzania. Bull World Health Organ.

[7] Ahmed HM, Mitchell M, Hedt B (2010). National implementation of Integrated Management of Childhood Illness (IMCI): policy constraints and strategies. Health Policy.

[8] Walter ND, Lyimo T, Skarbinski J, Metta E, Kahigwa E, Flannery B, Dowell SF, Abdulla S, Kachur SP (2009). Why first-level health workers fail to follow guidelines for managing severe disease in children in the Coast Region, the United Republic of Tanzania. Bull World Health Organ.

[9] WHO, Development. NFfS (2007). Integrated Management of Childhood illness Computarized Adaptation and Training tool (ICATT).

[10] DeRenzi B, Lesh N, Parikh TS, Sims C, Maokola W, Chemba M, Hamisi Y, Schellenberg D, & Borriello G (2008, April). E-IMCI: improving pediatric health care in low-income countries. ACM Conference on Computer-Human Interaction (CHI).

[11] Mitchell M, Lesh N, Cranmer H, Fraser H, Haivas I, Wolf K (2009). Improving care - improving access: the use of electronic decision support with AIDS patients in South Africa. Int J Healthcare Technology and Management.

[12] Mitchell M, Hedt BL, Eshun-Wilson I, Fraser H, John MA, Menezes C, Grobusch MP, Jackson J, Taljaard J, Lesh N (2012). Electronic decision protocols for ART patient triaging to expand access to HIV treatment in South Africa: a cross sectional study for development and validation. Int J Med Inform.

[13] Mitchell M, Getchell M, Nkaka M, Msellemu D, Van Esch J, Hedt-Gauthier B (2012). Perceived improvement in integrated management of childhood illness implementation through use of mobile technology: qualitative evidence from a pilot study in Tanzania. J Health Commun.

[14] Wang SJ, Lengeler C, Mtasiwa D, Mshana T, Manane L, Maro G, Tanner M (2006). Rapid Urban Malaria Appraisal (RUMA) II: epidemiology of urban malaria in Dar es Salaam (Tanzania). Malar J.

[15] D’Acremont V, Kahama-Maro J, Swai N, Mtasiwa D, Genton B, Lengeler C (2011). Reduction of anti-malarial consumption after rapid diagnostic tests implementation in Dar es Salaam: a before-after and cluster randomized controlled study. Malar J.

[16] Suri H (2011). Purposeful Sampling in Qualitative Research Synthesis. Qualitative Research Journal.

[17] Adam J, Kamuzora F (2008). Research Methods for Business and Social Studies. Mzumbe Book Project.

[18] Mayring P. Qualitative Content Analysis [28 paragraphs]. Forum Qualitative Sozialforschung / Forum: Qual Soc Res. 2000;1(2):Available at http://www.qualitative-research.net/index.php/fqs/article/view/1089/2385 Accessed in April 2013.

[19] Nyamtema AS (2010). Bridging the gaps in the Health Management Information System in the context of a changing health sector. BMC Med Inform Decis Mak.

[20] Moxey A, Robertson J, Newby D, Hains I, Williamson M, Pearson SA (2010). Computerized clinical decision support for prescribing: provision does not guarantee uptake. J Am Med Inform Assoc.

[21] Mitchell M, Hedt-Gauthier BL, Msellemu D, Nkaka M, Lesh N (2013). Using electronic technology to improve clinical care – results from a before-after cluster trial to evaluate assessment and classification of sick children according to Integrated Management of Childhood Illness (IMCI) protocol in Tanzania. BMC Med Inform Decis Mak.

[22] Adam T, Manzi F, Schellenberg JA, Mgalula L, de Savigny D, Evans DB (2005). Does the Integrated Management of Childhood Illness cost more than routine care? Results from the United Republic of Tanzania. Bull World Health Organ.

[23] Baiden F, Owusu-Agyei S, Bawah J, Bruce J, Tivura M, Delmini R, Gyaase S, Amenga-Etego S, Chandramohan D, Webster J (2011). An evaluation of the clinical assessments of under-five febrile children presenting to primary health facilities in rural Ghana. PLoS One.

[24] Horwood C, Vermaak K, Rollins N, Haskins L, Nkosi P, Qazi S (2009). An evaluation of the quality of IMCI assessments among IMCI trained health workers in South Africa. PLoS One.

[25] Bryan L, Garg R, Ramji S, Silverman A, Targa E, Ware I. Investing in Tanzania Human Resources for Health: an HRH report for the TOUCH Foundation, Inc, McKinsey & Company. July 2006.

[26] Munga MA, Maestad O (2009). Measuring inequalities in the distribution of health workers: the case of Tanzania. Hum Resour Health.

[27] Dominic A, Kurowski C (2005). Human Resources for Health – an Appraisal of the Status quo in Tanzania Mainland.

[28] Maestad O, Torsvik G, Aakvik A (2010). Overworked? On the relationship between workload and health worker performance. J Health Econ.

[29] Paul F. Health Worker Motivation and the Role of Perfomance Based Finance System in Africa: A Qualitative Study on Health Worker Motivation and the Rwandan Perfomance Basel Finance Initiative in District Hospitals. LSE Department of International Development (ID), London, United Kingdom .2009;2008(96).

[30] Leshabari MT, Muhondwa EPY, Mwangu MA, Mbembati NAA (2008). Motivation of health care workers in Tanzania: a case study of Muhimbili National Hospital. East African Journal of Public Health.

[31] Hor CP, O’Donnell JM, Murphy AW, O’Brien T, Kropmans TJ (2010). General practitioners’ attitudes and preparedness towards Clinical Decision Support in e-Prescribing (CDS-eP) adoption in the West of Ireland: a cross sectional study. BMC Med Inform Decis Mak.

[32] Maestad O, Mwisongo A (2011). Informal payments and the quality of health care: Mechanisms revealed by Tanzanian health workers. Health Policy.

[33] Fossum M, Ehnfors M, Fruhling A, Ehrenberg A (2011). An evaluation of the usability of a computerized decision support system for nursing homes. Appl Clin Inform.

[34] Sedlmayr B, Patapovas A, Kirchner M, Sonst A, Muller F, Pfistermeister B, Plank-Kiegele B, Vogler R, Criegee-Rieck M, Prokosch HU (2013). Comparative evaluation of different medication safety measures for the emergency department: physicians’ usage and acceptance of training, poster, checklist and computerized decision support. BMC Med Inform Decis Mak.

[35] WHO (2010). Key components of a well functioning health system.

[36] Leonard KL, Masatu MC, Vialou A. Getting Clinicians to Do Their Best: Ability, Altruism and Incentives. National Science Foundation, World Bank 2005:38.

